# Focal brain inflammation and autism

**DOI:** 10.1186/1742-2094-10-46

**Published:** 2013-04-09

**Authors:** Theoharis C Theoharides, Shahrzad Asadi, Arti B Patel

**Affiliations:** 1 Molecular Immunopharmacology and Drug Discovery Laboratory, Department of Molecular Physiology and Pharmacology, Tufts University School of Medicine, Suite J304, 136 Harrison Avenue, Boston, MA 02111, USA; 2 Department of Pharmacy, Tufts Medical Center, Boston, MA 02111, USA; 3 Department of Biochemistry, Tufts University School of Medicine, Boston, MA, USA; 4 Department of Internal Medicine, Tufts University School of Medicine and Tufts Medical Center, Boston, MA, USA; 5 Department of Psychiatry, Tufts University School of Medicine and Tufts Medical Center, Boston, MA, USA; 6 Graduate Program in Biochemistry, Sackler School of Graduate Biomedical Sciences, Tufts University, Boston, MA, USA

## Abstract

Increasing evidence indicates that brain inflammation is involved in the pathogenesis of neuropsychiatric diseases. Autism spectrum disorders (ASD) are characterized by social and learning disabilities that affect as many as 1/80 children in the USA. There is still no definitive pathogenesis or reliable biomarkers for ASD, thus significantly curtailing the development of effective therapies. Many children with ASD regress at about age 3 years, often after a specific event such as reaction to vaccination, infection, stress or trauma implying some epigenetic triggers, and may constitute a distinct phenotype. ASD children respond disproportionally to stress and are also affected by food and skin allergies. Corticotropin-releasing hormone (CRH) is secreted under stress and together with neurotensin (NT) stimulates mast cells and microglia resulting in focal brain inflammation and neurotoxicity. NT is significantly increased in serum of ASD children along with mitochondrial DNA (mtDNA). NT stimulates mast cell secretion of mtDNA that is misconstrued as an innate pathogen triggering an auto-inflammatory response. The phosphatase and tensin homolog (PTEN) gene mutation, associated with the higher risk of ASD, which leads to hyper-active mammalian target of rapamycin (mTOR) signalling that is crucial for cellular homeostasis. CRH, NT and environmental triggers could hyperstimulate the already activated mTOR, as well as stimulate mast cell and microglia activation and proliferation. The natural flavonoid luteolin inhibits mTOR, mast cells and microglia and could have a significant benefit in ASD.

## Introduction

### Focal brain inflammation

Increasing evidence indicates that brain inflammation is important in the pathogenesis of neuropsychiatric disorders [1,2]. Autism spectrum disorders (ASD) are pervasive neuro-developmental disorders characterized by varying degrees of deficiencies in social interactions, intelligence, and language, as well as the presence of stereotypic behaviors [3-6]. Recent results from the Centers of Disease Control in the USA indicate that as many as 1/80 children have ASD [7]. Many such children regress at about age 3 years, often after a specific event such as reaction to vaccination, infection [8,9], trauma [10,11], toxic exposures [12] or stress [13], implying the importance of some environmental triggers [14,15].

Increasing evidence points to some immune dysfunction/inflammation in ASD [16,17]. The markers of inflammation identified in the brain and cerebrospinal fluid (CSF) of many ASD patients include TNF, IL-6 and monocyte chemotactic protein 1 (MCP-1), the latter of which also is chemotactic for mast cells [18]. Pro-inflammatory cytokine mRNA (IL-1α, IL-1β, IL-6 and TNF-α) is increased in brain inflammation and has been associated with hippocampal and cerebral damage [8]. Mast cells are a rich source of IL-6 and TNF [19]. In fact, mast cells are the only immune cells that store pre-formed TNF and can release it rapidly upon stimulation [20].

Mast cells and cytokines such as IL-6 and TNF are also implicated in disruption of the blood–brain barrier (BBB) [21-23], which may be malfunctioning or *leaky* in ASD as evidenced by the presence of circulating auto-antibodies directed against the fetal brain proteins [24-27]. We had reported that the cytokine IL-33 synergizes with inflammatory neuropeptides to stimulate mast cells and result in increased vascular permeability [28]. IL-33 has been considered an alarmin, acting through mast cells to alert the innate immune system [29,30], and has recently been linked to brain inflammation [31-33].

We have also reported that neurotensin (NT) and corticotropin-releasing hormone (CRH), secreted under stress, synergistically stimulate mast cells, leading to increase vascular permeability [34] and contribute to BBB disruption [35]. We further showed that NT stimulates mast cell secretion of vascular endothelial growth factor (VEGF) [36], which is also vasodilatory. NT also increases expression of CRH receptor-1 (CRHR-1) [37], activation of which by CRH increases allergic stimulation of human mast cells [38].

NT is a vasoactive peptide originally isolated from the brain [39], but also found in the gut where it has been implicated in inflammation [40], and in increased intestinal permeability in rodents [41]. NT is also increased in the skin following acute stress, stimulates skin mast cells and increases vascular permeability in rodents [42]. NT stimulates rodent peritoneal mast cells to secrete histamine and elevates histamine plasma levels through activation of specific NT receptors (NTR) [43-45]. Moreover, NT is rapidly degraded by mast cell proteases [34,46] implying tight regulation of its activity.

Mast cells are hemopoietic-derived tissue immune cells responsible for allergies, but also implicated in immunity [47] and inflammation [18]. Mast cells can produce both pro- and anti-inflammatory mediators [48] and may have immuno-modulatory functions [47,49-51]. It is, therefore, of interest that allergic-like reactions are common in ASD children [52,53] implying activation of mast cells by non-allergic triggers [17]. The richest source of mast cells in the brain is the diencephalon [54] that regulates behavior, while the highest concentration of NTR is in the Broca area [55], which regulates language, known to be lost in many children with ASD. Mast cells are responsible for eliciting neutrophil infiltration that promotes inflammation [56]. Mast cell-microglial interactions are important in neuroinflammatory diseases [57,58]. Microglia are the innate brain immune cells that are increasingly implicated in a number of neuropsychiatric diseases [59]. In fact, abnormal microglial growth and activation was recently reported in the brain of ASD patients [60,61]. Microglia express NTR3, activation of which leads to their proliferation [62].

**Figure 1:**
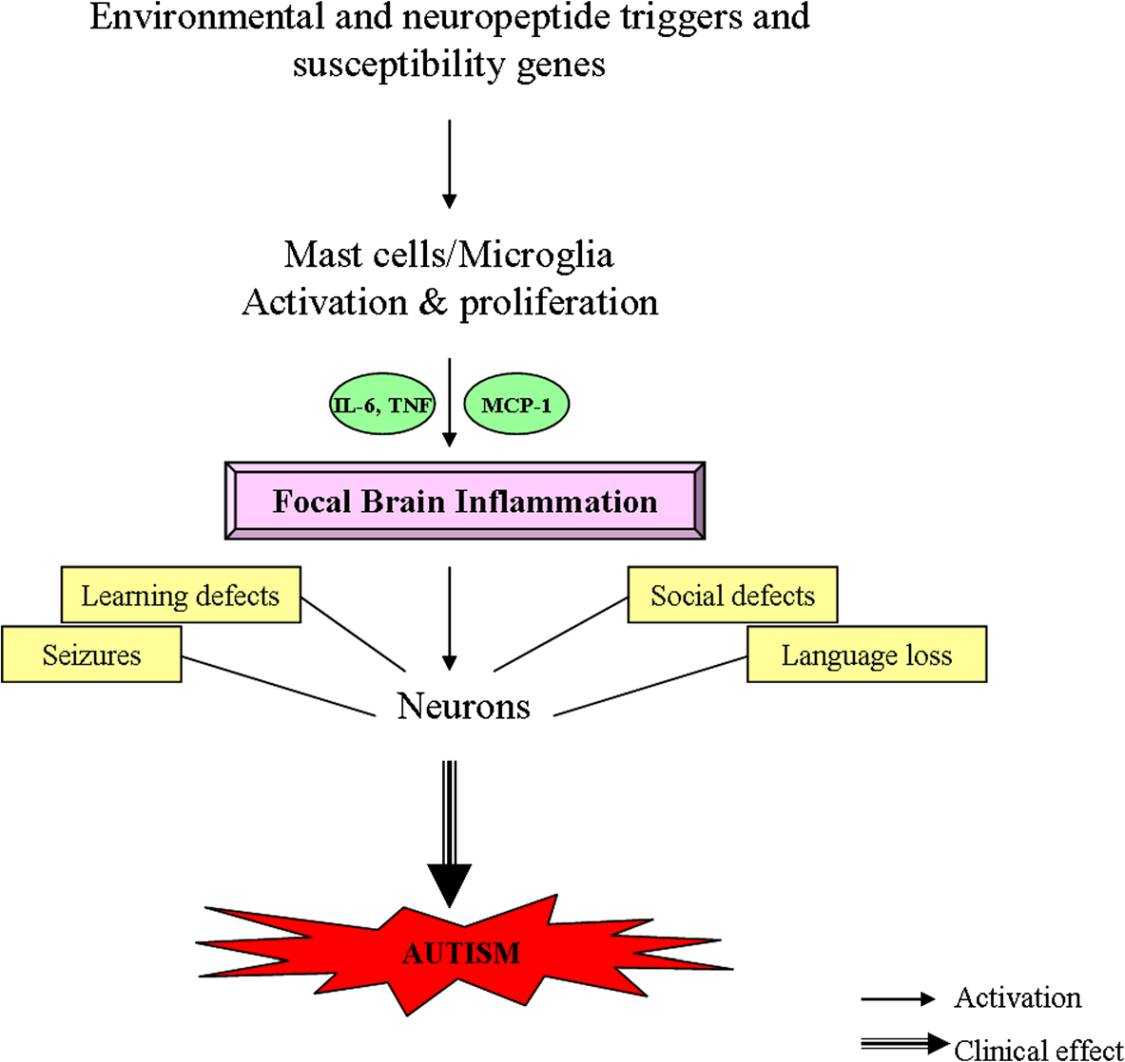
**Diagrammatic representation of how stimulation of mast cells and microglia could lead to multiple effects that contribute brain inflammation and the pathogenesis and symptoms of autism.** MCP, monocyte chemotactic protein.

NT has additional actions that are relevant to ASD (Table 1): it induces intestinal secretion and mobility [63], stimulates glial cell proliferation [64], and can facilitate seizures through activation of glutamate receptors [65]. In fact, the glutamate receptor mGluR5 was reported to be overactive in fragile X mice [66,67], a condition associated with high risk of ASD. In other words, NT could contribute to ASD pathogenesis through different mechanisms (Figure 1).

**Table 1:** Neurotensin actions relevant to autism spectrum disorder (ASD) pathogenesis

**Effect**	**Result**
Activation and proliferation of microglia	Brain inflammation
Activation of mast cells	Blood–brain-barrier disruption and inflammation
Disruption of gut-blood barrier	*Leaky gut* and inflammation
Mast cell stimulation, especially in the subgroup of ASD patients with allergic symptoms	Augmentation of allergic symptoms
Extracellular secretion of mitochondrial components that act as innate pathogens	Inflammation
Stimulation of glutamate receptors	Neuronal damage
Direct neurotoxicity	Neuronal damage

There is also support for increased oxidative stress [68] and some mitochondrial (mt) defects at least in subgroups of patients with ASD [69]. We showed that mtDNA is significantly increased in the serum of young autistic children [70], who also had significantly increased serum level of NT [71]; this triggers mast cells to secrete mtDNA [38] that acts as innate pathogen to stimulate mast cells [72] and other immune cells, leading to auto-inflammation [73]. Moreover, mtDNA can cause neuronal degeneration and altered behavior [74]. We believe that ASD originate from immune perinatal insults [75,76] that activate ASD susceptibility genes leading to focal encephalitis (Table 2).

**Table 2:** Key pathologic processes in ASD^*^

**Change**	**Pathologic processes**
↑	Allergic-like symptoms
↑	Anti-brain protein auto-antibodies
↑	Food intolerance
↑	Brain and gut inflammatory markers
↑	High anxiety and response to stress
↑	Oxidative stress
↓	Glutathione
↓	Methylation, sulfation

^*^Not present all ASD children.

### Epigenetic activation of ASD susceptibility genes

In spite of the fact that almost 100 gene mutations have been identified in patients with ASD [77,78], they do not explain more than a few percent of ASD cases [6]. High risk for developing ASD has been associated with mutations leading to decreased phosphatase and tensin homolog (PTEN) and tuberous sclerosis protein 1 and 2 (TSC1/2) [77]. These proteins are upstream inhibitors of the mammalian target of rapamycin (mTOR) [77,79], which leads to microglia and mast cell proliferation [80,81]. Activation of susceptibility genes is being increasingly invoked to explain ASD [7,82]. A recent paper reported that offspring of maternal immune activation in mice led to increased IL-6 and IL-17, and contributed to ASD-related behaviors [9]; repopulation of control irradiated mice with bone marrow derived from affected mothers did not induce those effects suggesting the contribution of some epigenetic environmental influences. Stimulation of mTOR in subjects with overactive mTOR due to gene mutations, leading to low PTEN, would contribute to a form of epigenetic signal.

### Novel treatments

Behavioral interventions are the most common treatment approaches [83], but do not address the core ASD symptoms [84,85]. Psychotropic drugs are used much too often in ASD [86-88]. Such drugs include antipsychotic medications [89], the newer atypical compounds [90,91] risperidone [92,93] and aripiprazole [94] for obsessive-compulsive symptoms, aggression and self-injury, as well as methylphenidate for hyperactivity [95]. However, two recent reviews concluded that there is insufficient evidence to support any benefit of psychotropic drugs [96] or selective serotonin re-uptake inhibitor (SSRIs) [97] in ASD. In fact, the SSRI citalopram may actually be detrimental [98], especially in children [99]. Moreover, a recent paper reported that citalopram administration perinataly altered cortical network function and led to ASD-like behaviors in rodents [100].

**Figure 2:**
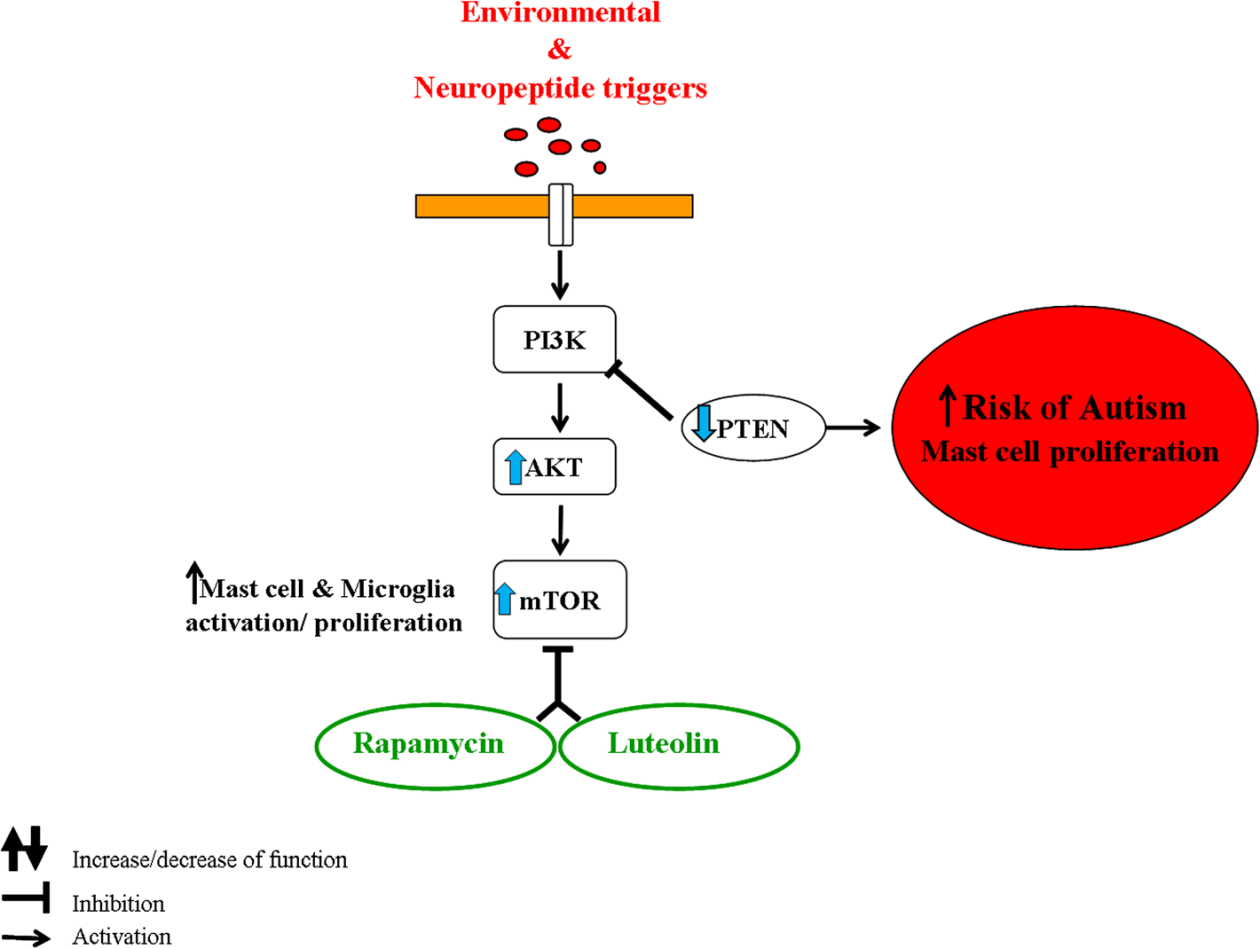
**Diagrammatic representation of the mTOR pathway, how it may lead to increased risk of autism and the inhibitory effect of luteolin.** mTOR, mammalian target of rapamycin; PTEN, phosphatase and tensin homolog; AKT, protein kinase B.

Rapamycin and its analogs are mTOR inhibitors [101] and are being tried for treatment of ASD [102-105] (Figure 2). Our preliminary results (not shown) indicate for the first time that the natural flavonoid luteolin [106] is more potent that rapamycin in its ability to inhibit human mast cell TNF release (Figure 2). A previous report also indicated that flavonoid-related epigallocatechin gallate (EGCG) is an mTOR inhibitor [107]. Luteolin may not only inhibit mTOR, but also has additional beneficial effects in brain inflammation. It inhibits oxidative stress [106], inflammation [106], mast cell degranulation [108], mast cell cytokine release [38], thimerosal-induced inflammatory mediator release [109], microglial activation and proliferation [110-112], and auto-immune T cell activation [113,114]. Luteolin is also protective against methylmercury-induced mitochondrial damage [115], is neuroprotective [116] and mimics brain-derived neurotrophic factor (BDNF) [117], which was recently associated with autistic-like-behavior in mice [118]. Finally, luteolin could reverse ASD-like behavior in mice [53], and was recently shown to have significant benefit in children with ASD [38,119].

## Conclusions

The prevalence of ASD continues to rise, but there is no clinically effective drug for the core ASD symptoms. Unfortunately, the lack of distinct pathogenesis and biomarkers makes it difficult to develop effective treatments. Stimulation of mTOR, which is already activated due to PTEN mutations, by NT, CRH and/or IL-33, could serve as novel targets for drug development. NTR and CRHR-1 antagonists could, therefore, be used in ASD, along with luteolin.

## Abbreviations

ASD: autism spectrum disorders; BBB: blood–brain barrier; BDNF: brain-derived neurotrophic factor; CSF: cerebrospinal fluid; CRH: corticotropin-releasing hormone; EGCG: epigallocatechin gallate; IL: interleukin; MCP: monocyte chemotactic protein; mTOR: mammalian target of rapamycin; mt: mitochondrial; NT: neurotensin; NTR: neurotensin receptor; PTEN: phosphatase and tensin homolog; SSRI: selective serotonin re-uptake inhibitor; TNF: tumor necrosis factor; TSC1/2: tuberous sclerosis protein 1 and 2; VEGF: vascular endothelial growth factor.

## Competing interests

The authors declare they have no competing interests.

## Authors’ contributions

TCT, SA, and AP conceived and wrote the manuscript. All authors read and approved the final manuscript.
